# Inflammatory Myofibroblastic Tumors in the Uterus: Childhood-Case Report and Review of the Literature

**DOI:** 10.3389/fped.2020.00036

**Published:** 2020-02-14

**Authors:** Péter Etlinger, Levente Kuthi, Tamás Kovács

**Affiliations:** University of Szeged, Szeged, Hungary

**Keywords:** inflammatory, myofibroblastic, tumor, uterus, children

## Abstract

Inflammatory myofibroblastic tumor (IMT) is a spindle cell neoplasm with low malignant potential, which may appear in different parts of the body. Uterine localization is rare, especially among children. Etiology is unclear, although some authors suggest underlying trauma or distress. A 3.5-year-old girl was treated at our institute for recurring vaginal bleeding without injury or known pathology. Physical examination and laboratory analysis revealed no specific findings, contrast-enhanced MRI found a 25 × 28 × 30 mm-sized inhomogeneous soft tissue mass in the uterus wall, which was excised in toto. Histological examination identified a spindle cell pattern, and the FISH test revealed ALK gene rearrangement, the lesion was defined as an IMT. Six cases were published to date, and their diagnostic methods are not equivocal, CT, and PET CT were preferred instead of MRI. Aggressive therapy seems to be exaggerated according to low recurrence and metastasis occurrence, and crizotinib is proved as good therapeutic agent in those cases. Biopsy and histology has important role in order to distinguish IMT from malignancies completed with FISH examination because ALK positivity strengthens the diagnosis. No lethal outcome was published among children, as our patient is also symptom-free after 3 years.

## Background

In the WHO definition, inflammatory myofibroblastic tumor (IMT) is a rare spindle cell neoplasm with low malignant potential, and it is composed of proliferative myofibroblasts and mixed inflammatory cell infiltrate. The appearance of IMT in childhood is extremely rare. Symptoms and treatment are heterogeneous, mainly depend on the localization of the tumor (1–4). Genetically speaking, approximately half of IMTs harbor clonal rearrangement of the anaplastic lymphoma kinase (*ALK*) gene, which encodes receptor tyrosine kinase (1–5). Here, the authors present a uterine-located IMT case and review the current literature data.

## Case Report

**Figure 1:**
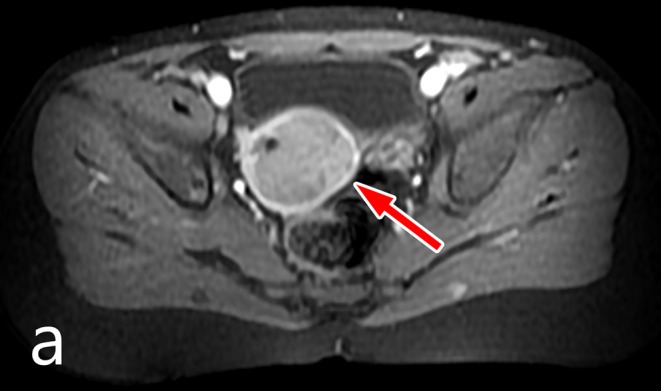
MRI image of the uterine lesion.

A 3.5-year-old girl was referred to the surgical outpatient clinic complaining of recurring vaginal bleeding. There was no trauma or inflammatory symptoms in her clinical history. Regarding the family history, no malignant, or genetic disease was noted. On physical examination flat and non-tender abdomen was found without any palpable lumps, with negative rectal examination. Abdominal ultrasound scan showed a mildly enlarged uterus, while MRI examination identified a 25 × 28 × 30 mm sized inhomogeneous soft tissue mass in the uterus that compressed the entire endometrium (Figure 1). The lesion enhanced contrast material, and it did not spread through the serosa. AFP and LDH values were in normal range, but NSE level was mildly elevated.

According to the multidisciplinary team's decision, primary open surgery was chosen. Fine needle biopsy was excluded because of pelvic localization, small size, and the chance of inadequate sample size for a certain histologic opinion. Tumor spilling was also considered as risk factor during this procedure. Surgery was performed through suprapubic incision; and the well-circumscribed lesion seemed resectable, so instead of tumor biopsy the entire mass was removed from the posterior wall of the uterus. The myometrium was completely reconstructed. Lymph node metastasis was not found. The postoperative period was uneventful. After a 3-years follow-up period the child was complaint-free, and the abdominal US scans showed no recurrence. Histological examination revealed IMT with irregular borders composed of spindle cells with low cytological atypia. Also, an extensive inflammatory cell infiltrate with eosinophyl granulocytes and lymphocytes was observed in the tumor tissue. Immunohistochemistry was positive for h-caldesmon, smooth muscle actin, CD34, factor XIII and ALK. ALK FISH examination has found ALK translocation in 80% of the tumor cells.

## Discussion

IMTs in children are seldom found, especially in uterine localization (6). It usually appears in the lung or liver, but some reports also mentioned other possible sites such as the stomach (7), mesentery, omentum, retroperitoneum, head and neck region, minor pelvis, urogenital tract, as well as extremities (2, 4). Symptoms vary depending on the localization, e.g., palpable mass, bleeding and vomiting, or bowel obstruction in abdominal located IMTs (8). Etiology is unknown, however reports suggest probability of previous injury, inflammation, or distress (8). In the Pubmed^©^ database only 6 reports concern pediatric uterine IMT cases (6, 9–12). The spectrum of their clinical findings varied from asymptomatic to severe physical signs such as abdominal and pelvic pain (67%), weight loss (40%), fever (40%), and menorrhagia (20%) (see Table 1). Tumor size ranged between 8 and 20 cm, while in our case maximum diameter was only 2.8 cm.

**Table 1:** Literature data of pediatric IMTs.

**References**	**Age (years)**	**Symptom**	**Location**	**Extent**	**Treatment**	**Tumor size (cm)**	**Gross appearance**	**Spindle cell pattern**	**Tumor border**
Bennett et al. (6)	8	n/a	n/a	n/a	Excision	20	n/a	Compact	n/a
Rabban et al. (9)	6	Abdominal pain	Myometrium	Filled endometrial cavity	Hysterectomy	12	Flesh-like	Fascicles	Focally irregular
Rabban et al. (9)	14	Weight loss, fever	Myometrium	Extent to parametrium	Hysterectomy	n/a	Fibrous	Hyalinized nests	Focally irregular
Fraggetta et al. (10)	10	Menorrhagia, malaise, abdominal discomfort, pelvic pain	Endometrium	Prolapse to vagina	Hysterectomy	8	Polypoid mass	Fascicular pattern	n/a
Gupta et al. (11)	14	Abdominal pain, nausea, weight loss, fever	Myometrium	n/a	Excision	11	Trabecular	Embedded between inflammatory cells	n/a
Gilks et al. (12)	6	Abdominal pain, distension	Myometrium	Compressed endometrial cavity	Hysterectomy	12	Fleshy	Fascicles	Well-circumscribed

There is no single imaging method that is universally agreed upon; contrast-enhanced CT (11) and PET-CT (10) were also performed in different pediatric cases in order to detect activity and metastases. Our chosen diagnostic modality was MRI for its better soft tissue specificity and to avoid radiation exposure, especially in the ovarian region. Interestingly, this tool has not been used in pediatric uterine IMTs. Diagnostic laparoscopy was performed prior to surgery in one case (11).

All six cases were treated surgically, four by hysterectomy (9, 10, 12) and two by local tumor removal (6, 11). Depending on the size and myometrial infiltration of the tumor, especially in children, organ-sparing local excision was preferred; this was also performed in our case.

Lymph node metastasis was present in one case without progression during follow-up (10). One recurrence was observed (6). She had the first local recurrence 1 month after the surgery, although it could be the result of incomplete resection during local excision (data not available), followed by two (ovarian and unknown) recurrences treated successfully with crizotinib.

The overall recurrence rate for IMT is about 25%, while distant metastases are very uncommon (2%) (2). Biological behavior mostly depends on the size, mitotic activity, tumor cell necrosis and resection line positivity (3). As some authors also reported, recurrence can be the consequence of incomplete resection, however in those cases no further relapse was detected (6, 8).

The histological appearance of each varied from case to case. Immunohistochemistry revealed ALK-positivity in 4 cases as it was also seen in our presented case. The expression of ALK protein and the presence of ALK gene rearrangement are good diagnostic markers; however, they are only present in about 50% of the IMTs, but in 88–100% of uterine IMTs (4, 5, 13, 14). A variable ALK-positivity can be seen in other tumor subtypes, such as anaplastic large cell lymphoma, rhabdomyosarcoma, and neuroblastoma (13), but in the case of female genital tract tumors, it is considered as a specific marker of IMTs thus supporting diagnosis. ALK translocation also offers a therapeutic option, because ALK inhibitors like crizotinib and alectinib can deactivate uncontrolled cell proliferation. ALK inhibitor treatment is recommended in surgically incurable, metastatic and recurrent cases (13, 14).

The “wait-and-see” approach was also described as a safe treatment of IMTs in adults as well as in pediatric cases (8, 15, 16). Data were also published about spontaneous regression of a hepatic IMT (8). However, to distinguish uterine IMTs from highly malignant tumors like leiomyosarcoma, either a biopsy or an excision is recommended. In contrast with pediatric cases, the outcome is worse among adults. Two lethal outcomes and six recurrences in 59 adult patients with uterine IMT were found (6, 17).

In conclusion, the so far published youngest child with uterine IMT was treated successfully by our team. To date, no recurrence or metastasis has been observed. The authors would also like to stress the role of the uterus-sparing surgery to eliminate tumor or gain tissue specimen, and emphasize the importance of the prior MRI imaging instead of other reported methods in order to achieve recovery without harming the normal development of the female genital tract.

## Data Availability Statement

All datasets generated for this study are included in the article/supplementary material.

## Ethics Statement

This study was carried out in accordance with the recommendations of the Declaration of Helsinki with written informed consent from the parents. The protocol was approved by the scientific ethic committee of University of Szeged.

## Author Contributions

The study conception was performed and the manuscript was written by PE and TK. Data acquisition was by PE, LK, and TK. Revision was by LK and TK.

### Conflict of Interest

The authors declare that the research was conducted in the absence of any commercial or financial relationships that could be construed as a potential conflict of interest.

## Abbreviations

IMT: Inflammatory myofibroblastic tumor
ALK: Anaplastic lymphoma kinase
MDT: Multidisciplinary team
AFP: Alpha-fetoprotein
LDH: Lactate-dehydrogenase
NSE: Neuron-specific enolase
NPM: Nucleophosmin
FISH: Fluorescence *in situ* hybridization
TPM4: Trophomyosine 4
CLTC: Clathrin heavy chain.
